# Improving clinical named entity recognition in Chinese using the graphical and phonetic feature

**DOI:** 10.1186/s12911-019-0980-z

**Published:** 2019-12-23

**Authors:** Yifei Wang, Sophia Ananiadou, Jun’ichi Tsujii

**Affiliations:** 1 National Centre of Text Mining, University of Manchester, Manchester, UK; 2 Artificial Intelligence Research Center, National Research and Development Agency (AIST), Tokyo, Japan

**Keywords:** Text mining, Neural networks, Named entity recognition

## Abstract

**Background:**

Clinical Named Entity Recognition is to find the name of diseases, body parts and other related terms from the given text. Because Chinese language is quite different with English language, the machine cannot simply get the graphical and phonetic information form Chinese characters. The method for Chinese should be different from that for English. Chinese characters present abundant information with the graphical features, recent research on Chinese word embedding tries to use graphical information as subword. This paper uses both graphical and phonetic features to improve Chinese Clinical Named Entity Recognition based on the presence of phono-semantic characters.

**Methods:**

This paper proposed three different embedding models and tested them on the annotated data. The data have been divided into two sections for exploring the effect of the proportion of phono-semantic characters.

**Results:**

The model using primary radical and pinyin can improve Clinical Named Entity Recognition in Chinese and get the F-measure of 0.712. More phono-semantic characters does not give a better result.

**Conclusions:**

The paper proves that the use of the combination of graphical and phonetic features can improve the Clinical Named Entity Recognition in Chinese.

## Background

Named Entity Recognition (NER), as the name suggests, is a task to find the named entities from some given text. Named entities usually refer to some specific objects, such as persons and places. For the NER task in some languages using Latin alphabet like English, there are many available features to use, such as capital letters. But for Chinese, performing NER becomes difficult because there are no spaces between words and there are no capital letters to identify special words. Furthermore, we can get both semantic and phonetic information from English words, while Chinese characters in machines alone do not provide any information on them as they are just a sequence of Unicode. So Chinese character embedding containing both semantic and phonetic information should help in the NER task.

A radical is the basic graphical component to form the character. For example, , which means *illness*, has two radicals,  and . In this case,  is the primary radical and suggests the meaning of the character is related to illness, and  contains phonetic information suggesting the pronunciation of the character. The primary radical usually implies the meaning of a character. Table 1 shows some characters related to biomedicine with their meanings and primary radicals. It can be easily found that the names of a disease share the same primary radical and the names of organs share same primary radical as well. In the case of organs,  is the simplification form of .

**Table 1:** Phono-semantic characters in the biomedical domain

Character	Pinyin	Primary Radical	Phonetic Radical	Pinyin of Phonetic Radical
(illness)	bìng	(sickness)	(third)	bǐng
(tuberculosis)	láo	(sickness)	(labour)	láo
(pain)	tòng	(sickness)	(path)	yǒng
(liver)	gān	(moon)/(meat)	(do)	gàn
(chest)	xiōng	(moon)/(meat)	(ancient form of )	xiōng
(brain)	nǎo	(moon)/(meat)	(bad luck)	xiōng

Pinyin is a romanization system for Chinese, which can represent the pronunciation of a Chinese character in Latin letters. The pinyin of a character usually contains three parts: initial, final and tone. Initials and finals are similar to the consonant and vowel in English except there can be only one initial and one final in the pinyin of a character. There are five differny tones in the pinyin: flat tone ($\bar { }$), rising tone (´), falling-rising tone (ˇ), falling tone (`) and neural tone (). As shown in Table 1, the pinyin of  is bìng, where b is the initial, ing is the final and ì shows that the tone is falling tone. , the phonetic radical of  is also a Chinese character, whose pinyin is bǐng. In this example, only the tone is different. In some characters, such as  in Table 1, only the finals are the same in the original character and the phonetic radical, which are ong in this case. There is another case that the pinyin of the original character and the phonetic radical are completely different, but most characters sharing the same phonetic radical have similar pinyin. For example,  shown in Table 1 has the same pinyin with (nǎo) and (nǎo), although their phonetic radical  is pronounced as xiōng.

But not all Chinese characters present the meaning with their primary radical and the pronunciation with the phonetic radical. Chinese characters that have primary radicals and phonetic radicals are called phono-semantic characters, more than 90% of Chinese characters are phono-semantic [2]. Some examples of non-phono-semantic characters in biomedical domain are shown in Table 2.

**Table 2:** Non-phono-semantic characters in the biomedical domain

Character	Pinyin	Primary Radical	Other radical	Pinyin of Other Radical
(stomach)	wèi	(moon)/(meat)	(field)	tián
(heart)	xı̄n	(heart)	-	-
(harm)	hài	(roof)	(abundant) (mouth)	fēng kǒu

In Table 1, we can find that some of the biomedical characters are phono-semantic, containing primary radicals providing the semantic information and the phonetic radicals suggesting the pronunciation. So this paper attempts to explore whether primary radicals and the pinyin can help in Clinical NER in Chinese. While the characters in Table 2 do not have all the features, so applying the same method on these characters may not perform well, so another experiment is designed to explore how the proportion of phono-semantic characters will effect the result of using primary radicals and the pinyin.

Recently, with the development of deep learning, deep learning in Chinese NER has become popular. Wu et al. [3] applied the neural network with Conditional Random Field (CRF) to electronic health records and achieved the F-measure of 0.928. In the work of Peng et al. [4], word segmentation features were used to improve the Long Short-Term Memory-Conditional Random Field(LSTM-CRF) model and got the F-measure of 0.484 when tested on social media data.

In English, a subword has the similar feature of the radical in Chinese because it contains some semantic information and suggests the meaning of the word. Some research has been made on subwords in English. In the research of Luong et al. [5], the words were split into several subwords, which are usually prefixes, suffixes and word roots, and the embedding of each subword will be composed to get the embedding of the word. The work of Bojanowski et al. [6] uses n-gram as the subword and trains the embedding for subwords.

In Chinese, radicals and other graphical features have been used in embedding training. In the work of Yu et al. [7], a new embedding method named *JWE* is introduced. In *JWE*, all radicals are regarded as subwords and following *CBOW* [8] method. In the work of Cao et al. [9], *cw2vec* is introduced, where *word2vec* [8] is improved, strokes were used to form n-grams as subwords. Another try in NER is from the work of Dong et al., which uses character embedding and radical embedding to get a better performance [10]. There are also some other attempts to catch the graphical features contained in Chinese characters. For example, Dai et al. trains the characters into glyph embeddings [11].

## Methods

### Embedding models

In the work of processing subwords in both Chinese and English [6, 7, 9], the words are split into subwords first, and the subword embeddings are then trained to form the embedding of words. But when using primary radicals and pinyins, it is not a good idea to put them into one vocabulary to train because they are completely different things, thus it is meaningless to compare the similarity of primary radicals containing Chinese characters and pinyins containing Latin letters.

So the model proposed seeks to get the pretrained embeddings of primary radicals and pinyins separately and then combines them.

**Fig. 1:**
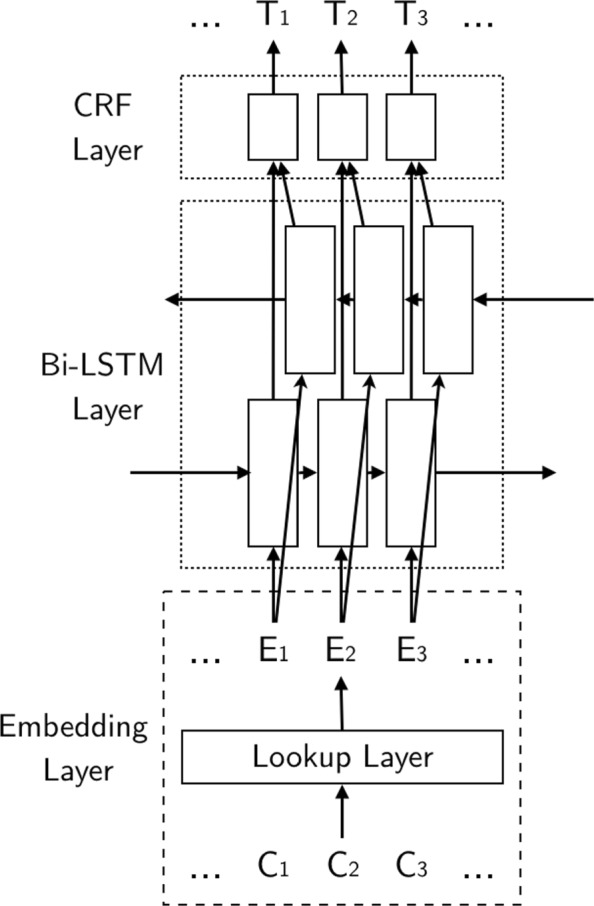
Bi-LSTM-CRF Model. The structure of Bi-LSTM-CRF model

In the normal method of using the neural network in Chinese NER, the character embedding will only contain its character. Figure 1 shows the structure of Bi-LSTM-CRF model [12]. In Fig. 1, *C*_*i*_ means the *i*th character, *E*_*i*_ means the embedding of the *i*th character, and *T*_*i*_ means the final tag of the character.

**Fig. 2:**
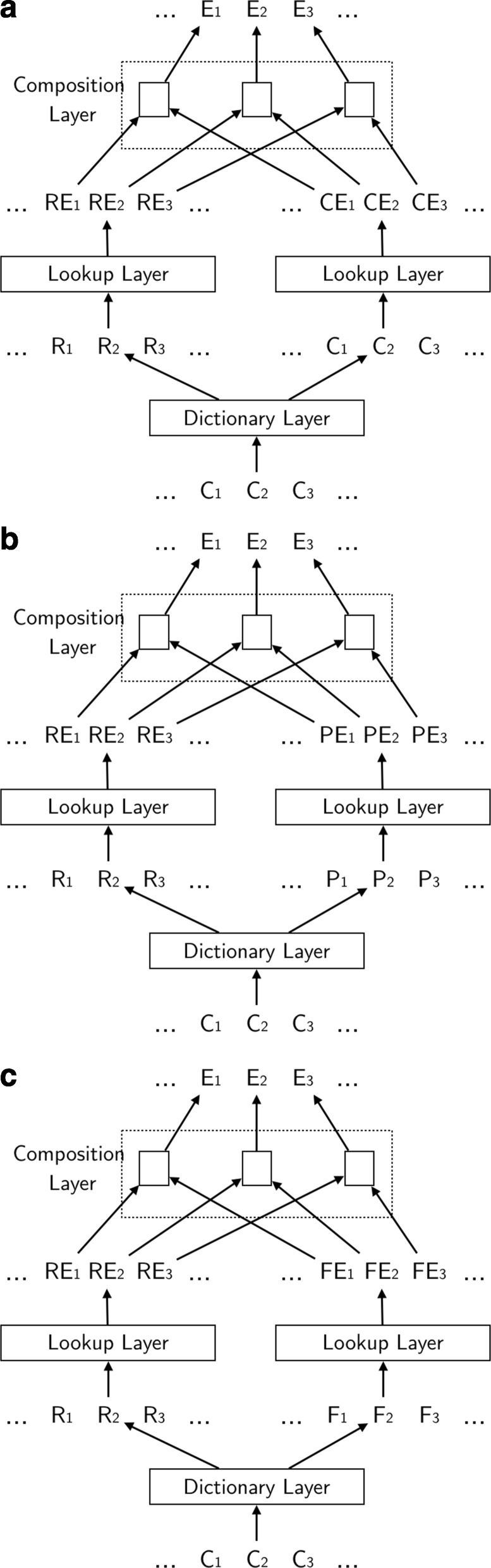
Proposed Models. The structures of proposed embedding models. **a** Radical+Character Model. **b** Radical+Pinyin Model. **c** Radical+Final Model

Different from simply looking for an embedding from the lookup layer, three different embedding models are proposed here to use the primary radical and other features in Fig. 2.

In the Radical+Character Model shown in Fig. 2a, the primary radical and the character itself are used to form the character embedding. The primary radical embedding *RE*_*i*_ and character embedding *CE*_*i*_ are obtained from the pretrained embeddings and form the final embedding *E*_*i*_ for character *C*_*i*_. This method is used to test how phonetic radicals affect the result.

In the Radical+Pinyin Model shown in Fig. 2b, the character embedding is formed by the primary radical and the pinyin. In Fig. 2b, *P*_*i*_, *PE*_*i*_ means the pinyin and pinyin embedding of character *C*_*i*_, respectively. Because phonetic radicals usually do not provide tone information, only the initial and final are used for the pinyin. This method considers both semantic radicals and phonetic radicals.

In the Radical+Final Model shown in Fig. 2c, the character embedding comes from the primary radical and the final of pinyin. *F*_*i*_ and *FE*_*i*_ represents the final of pinyin and the embedding of the final of pinyin, respectively. This model is a modified version of Radical+Pinyin Model, as the initial information sometimes is not provided by the phonetic radical.

In all models, there is a composition layer to combine two different embeddings. A method to combine embeddings is proposed below:

1 $$ E_{i}=LW_{i}*LE_{i}+RW_{i}*RE_{i}+b_{i}  $$

In Equation 1, *E*_*i*_ is the final embedding, *LE*_*i*_ and *RE*_*i*_ represent the embedding to be composed, *LW*_*i*_ and *RW*_*i*_ are weight matrices and *b*_*i*_ is the bias matrix. During the training, both weight matrices and bias matrices would be updated.

### Phono-semantic check

Currently, there is not a common way to know whether a Chinese character is phono-semantic or not. People usually believe a character is phono-semantic when they can think of some other characters sharing the same phonetic radical and similar pronunciation. *Shuowenjiezi*, the dictionary published in the early 2nd century, states how each Chinese character is built, where phono-semantic is one of the building method. But it is not a proper resource for checking a phono-semantic character, as there are great amount of characters built in later days by using phono-semantic method that are not included in this ancient dictionary.

Based on the feature of the phono-semantic character, one possible method to check whether a character is phono-semantic or not is to check the pinyin of the original character and all the pinyin of the forming radicals. If the original character shares the same final with one of the forming radicals, then the original character is a phono-semantic character.

However, there are some special cases. For example, the character  (tú) has two radicals,  and , where  is the primary radical containing semantic information, and the pinyin of  is zǒu. However,  is a phono-semantic character, whose phonetic radical is  (tǔ), one of the radicals of .

Ideographic Description Sequence (IDS) is a method to present how a Chinese-Japanaese-Korean (CJK) character is formed by using Ideographic Description Characters (IDC). For example, the IDS of  shown in Table 1 is , where the first character  is an IDC, suggesting how the following two characters are used to form the original character. In this case,  means that the first character should be on the top left of the second one. It is also possible to get the nested IDS of a Chinese character. In the case of ,  also has its IDS as , so that the nested IDS of  can be presented as . It is possible to find the phonetic radical via viewing all nested IDS.

The pseudo code of the method used to check whether a character is phono-semantic or not is shown in Algorithm 1. IDS() is the function to get the IDS of a character, if the character cannot be divided anymore, the character itself will be returned. Final() is the function to get the final of the pinyin of the character.


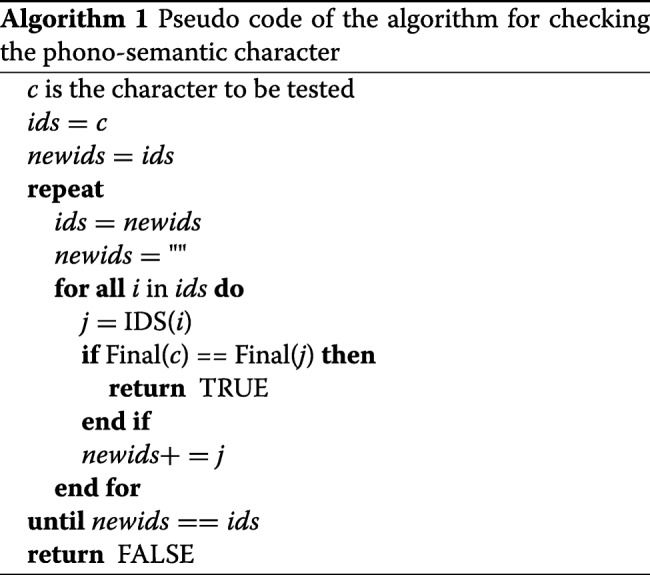


### Data

The data used in the experiment are provided in the China Conference on Knowledge Graph and Semantic Computing (CCKS) in 2017, which collects different clinical texts and contains 280,913 characters. The corpus uses BIO format to label five different named entity types: body part (BOD), symptom (SYM), disease (DIS), experiment (EXP) and treatment (TRE). A 5-cross-validation is performed to make the experiment.

To explore how the proportion of the phono-semantic characters affects the results, the data have been split into two sections. The percentages of the phono-semantic characters in each data are calculated. The data will be put in section A if the percentage is larger than the median. Otherwise, the data will be put in section B.

The details of two sections are shown in Table 3. The Phono-semantic% column shows the percentage of the phono-semantic characters in all characters, and The Unique Phono-semantic% column shows the percentage of the unique phono-semantic characters in all unique characters.

**Table 3:** Sections of data for phono-semantic test

	Phono-semantic%	Unique Phono-semantic%
A	36.6	46.2
B	33.0	43.9

The neural network model used for different embedding models is Bi-LSTM-CRF [12]. As only character embedding can be gained from the models proposed, the experiment here is done on character-based NER. The use of character-based NER will also prevent the problem of Chinese Word Segmentation.

To better catch the clinical texts, the pretrained embeddings are trained in a certain domain by using the Chinese Wikipedia under the category Medicine [13] and the category Biology [14] and their nested subcategories. Word2vec [15] package is used for pretraining character embedding, radical embedding and pinyin embedding.

To compare with other methods, the *JWE* model [7, 16] and the *cw2vec* model [9, 17] mentioned in Background part have also been tested. As both models are used for word embedding, each character in Wikipedia data is regarded as a word to train the character embedding. For a fair comparison, the same parameters including the learning rate, window size, embedding size have been used. The best model will be tested for the effect of the proportion of the phono-semantic characters.

The primary radical and pinyin information are gained from the Unihan database [18], IDS of all characters are gained from CHISE project [19], which annotates the IDS of most CJK characters.

## Results

### Model comparison

Table 4 shows the results of different models on CCKS data. R+C, R+P and R+F stand for the model in Fig. 2a, b and c, respectively. The model with (Sum) is a simplified version, where *LW*_*i*_ and *RW*_*i*_ are fixed as 1 and *b*_*i*_=0.

**Table 4:** F-measure of different models on CCKS data

Model	JWE	cw2vec	R+C	R+P	R+F	R+C(Sum)	R+P(Sum)	R+F(Sum)
BOD	0.614	0.595	0.666	0.688	0.661	0.660	0.678	0.652
SYM	0.716	0.682	0.724	0.746	0.735	0.725	0.734	0.727
DIS	0.623	0.528	0.677	0.777	0.629	0.666	0.751	0.574
EXP	0.706	0.666	0.715	0.720	0.723	0.721	0.710	0.721
TRE	0.516	0.513	0.672	0.618	0.549	0.667	0.616	0.519
ALL	0.669	0.635	0.696	**0.712**	0.695	0.696	0.702	0.687

The highest F-measure among all models is in bold

### Phono-semantic Proportion

Based on the result of model comparison, R+P model is used for exploring the affect of phono-semantic proportion. The result is shown in Table 5.

**Table 5:** F-measure of different sections using model R+P

	Section A	Section B
BOD	0.620	0.651
SYM	0.691	0.749
DIS	0.584	0.680
EXP	0.671	0.753
TRE	0.578	0.475
ALL	0.650	0.712

## Discussion

### Model comparison

In Table 4, it can be clearly found that Model Radical+Pinyin gives the best performance, especially on disease type. It is proved that the use of the graphical and phonetic feature of a character can be used for character embedding in an NER task as it has a better NER performance.

The reason that both JWE and cw2vec models do not have a good performance may be that they are designed for word embeddings. When applying them to character embedding, there might be some unnecessary operations and need more iterations for training.

The models that only sum two embeddings together have a slightly worse performance as *LW*_*i*_, *RW*_*i*_ and *b*_*i*_ are fixed, which is evidence that the learning process in the composition layer is necessary.

Tables 6 and 7 show the radicals occurring frequently in CCKS dataset. In both SYM and DIS types, there are some primary radicals occurring a lot, it might be the reason the model performs well in these two named entity types.

**Table 6:** Radical occurrence in named entities

BOD		SYM		DIS		EXP		TRE	
Radical	Occurrence	Radical	Occurrence	Radical	Occurrence	Radical	Occurrence	Radical	Occurrence
	17.2%		15.0%		11.0%		14.3%		8.1%
(meat)		(meat)		(meat)		(meat)		(water)	
	6.5%		13.0%		8.4%		5.5%		5.1%
(mouth)		(mouth)		(heart)		(wood)		(silk)	
	4.7%		10.4%		7.1%		5.3%		4.8%
(again)		(sickness)		(sickness)		(mouth)		(meat)	
	4.6%		4.9%		4.7%		5.1%		4.7%
(human)		(again)		(silk)		(human)		(grass)	
	4.1%		4.2%		4.6%		3.4%		3.7%
(state)		(big)		(fire)		(heart)		(hand)

**Table 7:** Overall radical occurrence in ccks data

Radical	Occurrence
(meat)	6.1%
(mouth)	4.9%
(wood)	3.8%
(human)	3.3%
(again)	3.2%

### Phono-semantic proportion

Based on the method how two sections are built and the phono-semantic percentage shown in Table 3, section A has more phono-semantic characters than section B does. If the model highly relies on phono-semantic characters, the model should have better result on section A. However, as shown in Table 5, the model performs better on section B.

Two sections have been reviewed, and new statistical result is shown in Table 8. The table is similar to Table 3, except that only named entity characters are considered. It shows that section B has more unique phono-semantic characters in named entities, which might be why the model has better performance on section B.

**Table 8:** Named entities in two sections

	Named Entity Phono-semantic%	Named Entity Unique Phono-semantic%
A	45.3	49.1
B	41.9	50.5

In both Tables 3 and 8, the proportion of phono-semantic characters is much smaller than the 90% stated by Boltz [2]. It is because the method for checking phono-semantic characters is not perfect yet, for example,  shown in Table 1 will not be considered as phono-semantic in this method. It might be another possible reason that such the result comes out.

It is also possible that the proportion of phono-semantic characters do not affect the performance of the embedding model. The reason that phonetic features can improve the result may be that a large amount of biomedical terms are translated with similar pronunciations, and models with phonetic features may be able to catch them. Some translated examples are shown in Table 9. More experiments are needed to find out the real reason.

**Table 9:** Translated biomedical terms

English	Chinese	Pinyin
Parkinson’s disease		pà jı̄n sēn shì zhèng
Aspirin		ā sı̄ pı̌ lín

## Conclusion

This work proposes a method to use the graphical feature and phonetic feature in Clinical NER in Chinese. Based on the experiment on Bi-LSTM-CRF, the model using the primary radical feature and pinyin can improve the performance. The F-measure has been improved by 0.043 when using model R+P compared to JWE.

But the work has the limitation that only character-based NER is tested, and some work should be done for word-based NER as well. It is also necessary to develop a better composition layer. A complex composition layer may result in good performance but require much more time for training, which is also a problem.

The results of exploring the affect of the proportion of the phono-semantic characters suggest that the new embedding model has better performance on lower proportion data. Some possible reasons are stated, and new experiments should be carried out to verify them.

## Data Availability

The ccks dataset analysed during the current study is adopted from the Chinese EMR NER task in China Conference on Knowledge Graph and Semantic Computing in 2017(ccks2017), but restrictions apply to the availability of these data,which were used under license for the current study, and so are not publicly available.

## Abbreviations

BOD: Body Part
CBOW: Continuous Bag-Of-Words
CJK: Chinese-Japanese-Korean
CRF: Conditional Random Field
DIS: Disease
EXP: Experiment
IDC: Ideographic Description Characters
IDS: Ideographic Description Sequence
JWE: Joint Learning Word Embedding
LSTM-CRF: Long Short-Term Memory-Conditional Random Field
NER: Named Entity Recognition
SYM: Symptom
TRE: Treatment
